# Influence of Different Biomaterials Extracted from Autologous Blood on the Cell Migration of Stem Cells from Dental Pulp

**DOI:** 10.3390/jfb16110398

**Published:** 2025-10-24

**Authors:** Janet N. Kirilova, Rositsa Z. Vladova, Viktoria P. Petrova, Sevda Yantcheva, Elitsa G. Deliverska, Nikolay D. Ishkitiev

**Affiliations:** 1 Department of Conservative Dentistry, Faculty of Dental Medicine, Medical University, 1000 Sofia, Bulgaria; r.vladova@fdm.mu-sofia.bg (R.Z.V.); victoria.petrova@fdm.mu-sofia.bg (V.P.P.); s.yancheva@fdm.mu-sofia.bg (S.Y.); 2 Department of Dental, Oral and Maxillofacial Surgery, Faculty of Dental Medicine, Medical University, 1000 Sofia, Bulgaria; 3 Department of Medical Chemistry and Biochemistry, Faculty of Medicine, Medical University, 1000 Sofia, Bulgaria; nischkitiev@medfac.mu-sofia.bg

**Keywords:** dental pulp stem cells, platelet concentrates, A-PRF, Solid PRF, scratch wound healing assay, dental pulp regeneration, tissue engineering

## Abstract

Background: This study aims to evaluate the effect of different types of platelet concentrates (autologous blood biomaterials) on the migration potential of human dental pulp stem (hDPSCs). Materials and Methods: Our team created a model of human dental pulp stem cells (hDPSCs). Various types of AB biomaterials were produced from blood samples from volunteers using the protocols presented: A-PRF+, Gel A-PRF+, and Solid PRF. The scratch wound healing assay was used to examine the closure of the experimental wounds on day 1 and day 14. The wound areas were quantified using Image J software. Statistical analysis was performed with the Kruskal–Wallis and Mann–Whitney U tests, as the data did not follow a normal distribution, which was confirmed by the Shapiro–Wilk test (*p* < 0.05). Results: The results demonstrate significantly faster closure of the experimental wounds on day 14 of the studied biomaterials AB: A-PRF+, Gel A-PRF+, and Solid PRF compared to the control group of cells. Gel A-PRF+ exhibited the most pronounced stimulatory effect on cell migration (*p* = 0.0036 vs. control), followed by Solid PRF and A-PRF+. Conclusions: The results indicate that autologous blood platelet concentrates stimulate the migration of hDPSCs in vitro. Gel A-PRF+ demonstrated the strongest effect, underscoring its potential clinical relevance for applications in tissue engineering.

## 1. Introduction

Regenerative endodontics has progressed from descriptive biology to clinically oriented strategies that leverage human dental pulp stem cells (hDPSCs) to promote reparative dentinogenesis and preserve pulp vitality. Recent studies demonstrate that early recruitment and directional migration of pulp progenitors to the exposure site are prerequisite steps that precede matrix deposition and dentin bridge formation, providing a mechanistic basis for vital pulp therapy. Emerging translational approaches—combining bioactive scaffolds, controlled growth-factor release, and minimally invasive protocols—aim to enhance the pulp–dentin complex’s innate healing potential and shorten recovery timelines [1].

From a patient perspective, vital pulp therapy offers tangible clinical benefits: it reduces treatment invasiveness, preserves sensibility and immune surveillance within the tooth, maintains structural integrity, and may lower the risk of long-term fractures relative to full pulpectomy. These advantages translate to fewer appointments, shorter chair time in selected cases, and improved long-term tooth survival, reduced need for root-canal therapy, particularly when indications are carefully selected and hemostasis and asepsis are rigorously controlled [2].

In the context of endodontic healing, the term “migration to damaged tissues” is used to denote the early recruitment of hDPSCs to the site of pulp exposure. This process is a necessary prerequisite for initiating subsequent reparative dentinogenesis, expressed by the formation of a hard-tissue dentin bridge, and for maintaining the vitality of the pulp’s soft-tissue component [1,2].

Accordingly, the ultimate therapeutic objectives of regenerative endodontics are twofold: (i) to induce and stabilize a hard-tissue response in the form of reparative dentin, establishing a biological barrier, and (ii) to preserve pulp vitality long-term with functional restoration and maintenance of the pulp–dentin complex. These parameters serve as clinically relevant endpoints for evaluating the effectiveness of biological and bioactive approaches, including the application of PRF biomaterials [1,2].

Seo BM and co-authors prove that human mesenchymal stem cells isolated from pulp, periodontal ligament, and bone marrow regenerate these tissues [3]. Human dental pulp stem cells (hDPSCs) have high proliferative capacity and multilineage differentiation potential. They have the ability to migrate and close tissue defects. This is essential for the regeneration of injuries in pulp tissues. Shi and Huang found that hDPSCs are promising for differentiation into odontoblast-like cells [4,5].

From human dental pulp mesenchymal stem cells in vitro conditions, a cell model can be created for conducting in vitro studies. This model is used to investigate the potential of various biological materials for repairing damage to the dental pulp. One such method is the scratch wound healing assay, which is used to study cell migration in two dimensions. The leading indicator for assessing wound healing is the rate at which the gap closes. It quantitatively reflects the dynamics of the collective movement of stem cells. The rate is determined by measuring the reduction in the cell-free area over time. By analyzing the results, the migration potential of biomaterials under different experimental conditions is compared [6].

Calcium–silicate cements, including MTA and Biodentine, constitute the benchmark for direct pulp capping because they ensure durable sealing, exhibit high biocompatibility, and consistently induce reparative dentin. In contrast, autologous PRF is positioned as a complementary bioactive adjunct that delivers a fibrin scaffold with sustained growth-factor release. Current evidence does not establish PRF as a full substitute for calcium–silicate cements; combined protocols remain a reasonable hypothesis pending rigorous clinical validation [7,8,9].

Platelet-rich fibrin (PRF) biomaterials are a second-generation platelet concentrate developed as a biologically more active material than platelet-rich plasma (PRP). Unlike PRP, the PRF manufacturing protocol does not use anticoagulants or exogenous thrombin, allowing for slow and natural fibrin polymerization and the formation of a three-dimensional network close to the physiological one. The PRF structure retains platelets and growth factors, which are released gradually, thus creating an autologous matrix that promotes cell migration, proliferation, and tissue regeneration [10,11,12]. Among the main factors identified in PRF are platelet-derived growth factor (PDGF), vascular endothelial growth factor (VEGF), transforming growth factor beta (TGF-β1), as well as interleukins (IL-1β, IL-6, IL-4) and tumor necrosis factor α (TNF-α), which together regulate the inflammatory response, angiogenesis, and stimulate cell migration [12]. PRF serves as a reservoir of bioactive molecules that stimulate wound healing and bone regeneration processes. Recent studies demonstrate the specific effect of PRF on pulp stem cell migration [13]. Margono et al. (2023) report that culturing these cells in the presence of Advanced PRF+ (A-PRF+) significantly increases their migration rate, confirming the therapeutic potential of PRF in tissue engineering [14].

Various protocols have been established for the preparation of autologous blood biomaterials, including A-PRF+, Gel A-PRF, and Solid PRF. However, despite their increasing use in regenerative dentistry, the comparative biological effects and clinical outcomes of these PRF variants in vital pulp therapy remain insufficiently investigated, and no comprehensive data are currently available in the scientific literature [15].

This investigation aims to evaluate the effect of different types of autologous blood biomaterials on the migration potential of hDPSCs using a scratch wound healing assay. The null hypothesis is that there is no difference in the stimulation of cell migration between the different types of biomaterials from autologous blood and the control conditions.

## 2. Materials and Methods

In accordance with the Helsinki Declaration II, a set of ethical principles for medical research involving human subjects, all subjects provided informed written consent for participation. The Medical University of Sofia Research Ethics Committee, a body responsible for ensuring the ethical conduct of research involving human participants, approved this project (approval code: 57 from 12 July 2024), a map for scientific developments and projects anticipating scientific research involving human participation. Ethical approval was obtained for the use of extracted human teeth, for venipuncture blood sampling, and for the conduct of clinical investigations.

The study was conducted on human dental pulp stem cell cultures. The cultures were prepared as follows:

### 2.1. Isolation and Cultivation of hDPSC

Human dental pulp samples were obtained from extracted third molars of healthy donors aged 18–25 years. The teeth were removed for orthodontic reasons under aseptic conditions. Third molars were impacted or partially erupted but not carious; teeth with prior endodontic treatment, restorations, cracks, or caries were excluded. After extraction, each tooth was cleaned of soft deposits and placed individually in separate sterile containers filled with DMEM (Dulbecco’s Modified Eagle Medium, high glucose; Sigma-Aldrich (St. Louis, MO, USA), Cat. No. D5648) and immediately transported to the laboratory under sterile conditions to ensure the preservation of cell viability. In the laboratory, each tooth was processed separately to avoid cross-contamination. The crown was carefully separated, and the dental pulp was extirpated with sterile endodontic instruments. The tissue fragments were incubated for 60 min at 37 °C in a solution containing Collagenase type I (Sigma-Aldrich, Cat. No. C0130) and Dispase II (Sigma-Aldrich, Cat. No. D4693), with periodic stirring. After centrifugation at 2700 rpm for 3 min, the cell pellet was resuspended in DMEM (Dulbecco’s Modified Eagle Medium, high glucose; Sigma-Aldrich, Cat. No. D5648).

The cells were cultured in DMEM, high glucose, enriched with 10% FBS (Fetal Bovine Serum, heat-inactivated; Sigma-Aldrich, Cat. No. F7524), 1% Antibiotic-Antimycotic solution (100×; Penicillin/Streptomycin/Amphotericin B; Sigma-Aldrich, St. Louis, MO, USA) and NaHCO_3_ (≥99.7%, Merck (Rahway, NJ, USA)/Sigma-Aldrich, Cat. No. A0384), at 37 °C, 5% CO_2_ and saturated humidity. The medium was replaced daily.

Upon reaching approximately 80% confluence, the cells were meticulously separated by treatment with 0.25% Trypsin-EDTA (Sigma-Aldrich, Cat. No. T4049) for 10 min, and then resuspended in PBS (Phosphate-Buffered Saline, pH 7.4; Sigma-Aldrich, Cat. No. P3813) and cultured in new Petri dishes. Human dental pulp stem cell cultures (passage P4) were seeded in 6-well plates and cultured until a confluent cell layer was reached within 24–48 h, ensuring the highest standards of cell culture.

To prove the human stem cell origin of the isolated hDPSCs, the cell cultures were stained immunofluorescently with the markers CD44 (HCAM, DF1485)—mouse monoclonal IgG1, 200 µg/mL (Santa Cruz Biotechnology, USA), Nestin (D-9)—mouse monoclonal IgG2a, 200 µg/mL (Santa Cruz Biotechnology, USA),CK19 (ab52625)—Cytokeratin 19, rabbit monoclonal, 0.838 mg/mL (Abcam, UK) and DSPP (LFMb-21)—mouse monoclonal IgG2b, 200 µg/mL (Santa Cruz Biotechnology, USA) and the nuclei were stained with DAPI (D9542)—nuclear fluorescent dye (Sigma-Aldrich, USA). The images were captured at ×60 magnification using IN Cell Analyzer 6000 system (GE Healthcare, Pittsburgh, PA, USA).

### 2.2. Preparation of Biomaterials from Autologous Blood (AB Biomaterial)

#### 2.2.1. Donor Inclusion and Exclusion Criteria

All participants provided written informed consent prior to blood collection. The inclusion criteria comprised clinically healthy ten male donors aged 25–35 years, non-smokers, with no history of systemic, infectious, or autoimmune diseases. The participants were not professional athletes and were not taking any medications known to affect platelet function (anticoagulants, corticosteroids, or nonsteroidal anti-inflammatory drugs).

The exclusion criteria included ongoing therapy with anticoagulant or anti-inflammatory drugs, the presence of acute infections, or hematological disorders.

Thirty milliliters of blood were collected from each participant via venipuncture (three 10 mL tubes). From these samples, three different protocols for AB biomaterials were used to prepare samples for each participant. The blood samples were centrifuged in two different centrifuges.

#### 2.2.2. Type of Centrifuges Used to Obtain Biomaterials from PRF

1. Centrifuge with fixed angle of the slots: DUO Quattro PRF centrifuge (Process for PRF, Nice, France) with the corresponding tubes provided according to the specifications of the same manufacturer with a red cap A-PRF (A-P by Choukroun, Process for PRF, Nice, France) and a green cap S-PRF (S Process for PRF, Nice, France) and are anticoagulant-free. The tubes differ in the internal treatment of their walls and are free from anticoagulants. The AB biomaterials A-PRF+ and Gel A-PRF+ were obtained according to the protocols of Choukroun J [10].
2. Centrifuge with horizontal arrangement of the slots during operation: Bio-PRF Horizontal Centrifuge with the corresponding tubes provided according to the specifications of the same manufacturer. Bio-PRF Red Glass Tubes, 100% glass (Bio-PRF, Jupiter, FL, USA). The test tubes differ in the internal treatment of their walls and are free of anticoagulants. The AB biomaterial Solid PRF was obtained according to Miron’s protocols [15].

A comparison of the technical characteristics of the two centrifuge types is presented in Table 1

Three groups of samples of tested AB biomaterials were created as follows:

1. Group 1. AB biomaterial Gel A-PRF+
2. Group 2. AB biomaterial Solid PRF
3. Group 3. AB biomaterial A-PRF+

#### 2.2.3. Protocols for AB Biomaterial: Gel A-PRF+; Solid PRF and A-PRF+

##### Protocol for Autologous Blood Biomaterial A-PRF+

Ten milliliters of venous blood, meticulously collected in red-capped A-P by Choukroun glass tubes, was used. The blood was then subjected to a precise centrifugation process in a DUO Quattro PRF centrifuge at 1300 rpm for 14 min. After this, the tubes were opened for 4–5 min, and the fibrin clot was separated from the erythrocyte mass with utmost care and precision, without cutting. The clot was then placed on a grid in a PRF box and compressed for 5–6 min until an A-PRF+ membrane was obtained, ensuring the highest quality of the biomaterial.

The amount of separated transudate collected with the processing box was used to prepare Gel A-PRF+.

##### Protocol for Autologous Blood Biomaterial Gel A-PRF+

Ten milliliters of venous blood, collected in green-capped S-PRF glass tubes, were used. The blood was centrifuged in a DUO Quattro PRF centrifuge at 1300 rpm for 14 min. After centrifugation, the upper liquid fraction, rich in platelets and fibrinogen, was separated with a sterile needle No. 18 and transferred to a sterile tray, where it was mixed with the transudate separated in the box to obtain A-PRF+. After 10–15 min, gelation occurs, and the resulting gel-like material is compressed six times with a hand tool to reduce the liquid and obtain a Gel-A-PRF+ membrane.

##### Protocol for Autologous Blood Biomaterial Solid PRF

Ten milliliters of venous blood, collected in Bio-PRF Red Glass Tubes, were used. The blood was centrifuged in a Bio-PRF Horizontal Centrifuge at 700 rpm for 8 min. After centrifugation, the tubes were opened for 4–5 min, after which the fibrin clot was separated from the erythrocyte mass without cutting. The clot was placed on a grid in a Bio-PRF box and compressed for 5–6 min until a Solid PRF membrane was formed.

Each membrane obtained according to the above protocols was weighed on an analytical scale KERN ABT 120-5DM (KERN & Sohn GmbH, Balingen, Germany) to determine the volume of the test materials. They were then placed in a test tube containing 10 mL of DMEM medium and incubated at 4 °C. The membranes remained in DMEM, and samples of the conditioned medium were collected on days 1 and 14.

### 2.3. Test Method: Scratch Wound Healing Assay

The scratch assay is a standardized surrogate of cell motility, modeling early recruitment relevant to initial closure of a pulp exposure; we acknowledge its limitations (cannot fully separate migration from proliferation) and cite its accepted use in dental tissue–engineering research. On the day before the experiment, the cell cultures were placed in a medium containing 1% FBS to minimize the effect of cell division. The cell cultures were placed in wells. A linear defect (“wound”) scratch zone was created in each well using a sterile tip of a 200 μL pipette. The wells were then washed three times with PBS (Phosphate-Buffered Saline, pH 7.4) to remove any detached cells.

1.8 mL of each type of AB biomaterial from was added to the prepared stem cell cultures for the first and second study periods. After the removal of 1.8 mL from the basal media for the first period of the experiment, an additional 1.8 mL of DMEM was added to the tubes containing the tested PRF membranes in order to restore the initial volume. After placing the conditioned medium in the wells, 0.2 mL of FBS was added to a final concentration of 10% to provide essential nutrients and growth factors for the cells.

**Figure 1:**
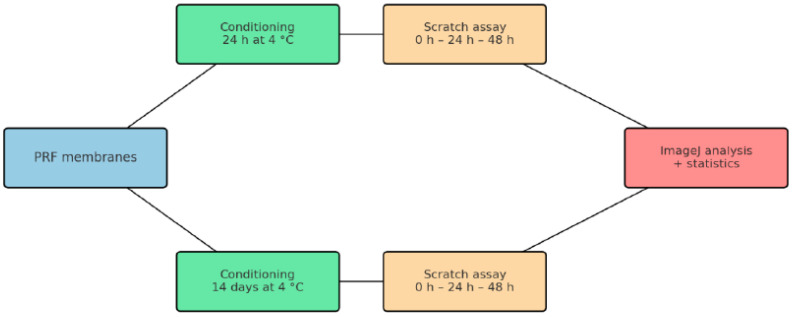
Schematic diagram of the experimental design. The PRF membranes were incubated in DMEM culture medium at 4 °C for two time periods: 24 h to observe the immediate effects, and 14 days to assess the long-term impact of the biomaterials on wound healing.

The scratch areas were photographed with an inverted phase-contrast microscope Leica DMI3000 B (Leica Microsystems, Wetzlar, Germany, objective 5×/0.12). immediately after the wound was created (0 h). The cultures were maintained at 37 °C, 5% CO_2_, and 50% humidity, and were documented again at 24 h and 48 h. The second experiment (14-day incubation of membrane samples) was performed on new cell cultures of pulp stem cells in 6-well plates with cells from passage P4 using the same Protocol (Figure 1).

The obtained images were analyzed using Wound Healing Size plugin for Image J (ImageJ v1.x/ Fiji distribution; Version 1.54p, National Institutes of Health, Bethesda, MD, USA) developed by Suarez-Arnedo et al. The plugin automatically distinguishes the wound area from the surrounding cell monolayer using contrast enhancement, variance filtering, and morphological reconstruction (“hole filling”) to isolate the open area. The analysis was carried out by measuring the wound area at each time point, which allowed for the accurate calculation of the wound closure rate, providing reliable and valid results for the study [16].

Advanced statistical methods were applied using IBM SPSS (Statistics 26, IBM, NY, USA), ensuring the robustness of the data analysis. Graphical representations were then created with Excel 2010, providing a clear and comprehensive visualization of the results. The normality of data distribution was assessed using the Shapiro–Wilk test, which indicated that the data did not follow a normal distribution. Therefore, non-parametric statistical tests were applied: the Kruskal–Wallis test for overall group comparisons and the Mann–Whitney U test with Bonferroni correction for pairwise analyses. A *p*-value of <0.05 was considered statistically significant. Effect sizes (r) and 95% CIs are reported.

## 3. Results

**Figure 2:**
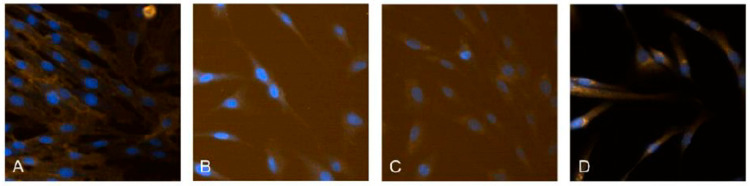
Immunofluorescence characterization of hDPSCs. Images with positive staining for (**A**) CD44, (**B**) Nestin, (**C**) CK19, and (**D**) DSPP. Nuclei are stained with DAPI. All images were captured at 60× magnification.

To confirm the phenotype of hDPSCs, immunofluorescence staining was performed (Figure 2), which showed positive expression of the mesenchymal marker CD44, confirming the mesenchymal origin of hDPSCs. The expression of Nestin and DSPP is consistent with their neurogenic and odontogenic potential, while CK19 highlights the possibility of epithelial-like differentiation and heterogeneity of the cell population.

The expression of the markers confirms the mesenchymal origin and multipotency of hDPSCs.

### 3.1. Results from the First Day of the Study

**Figure 3:**
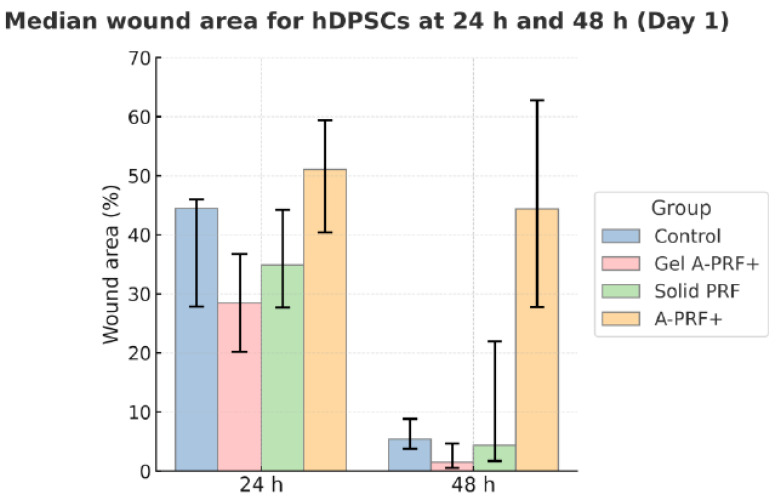
Scratch healing test—day 1. Quantitative analysis of wound area reduction (%) in hDPSCs at 24 h and 48 h after exposure to Control, Gel A-PRF+, Solid PRF, and A-PRF+. The chart illustrates the mean values showing the dynamics of wound closure on Day 1, with Gel A-PRF+ exhibiting the most pronounced effect.

**Figure 4:**
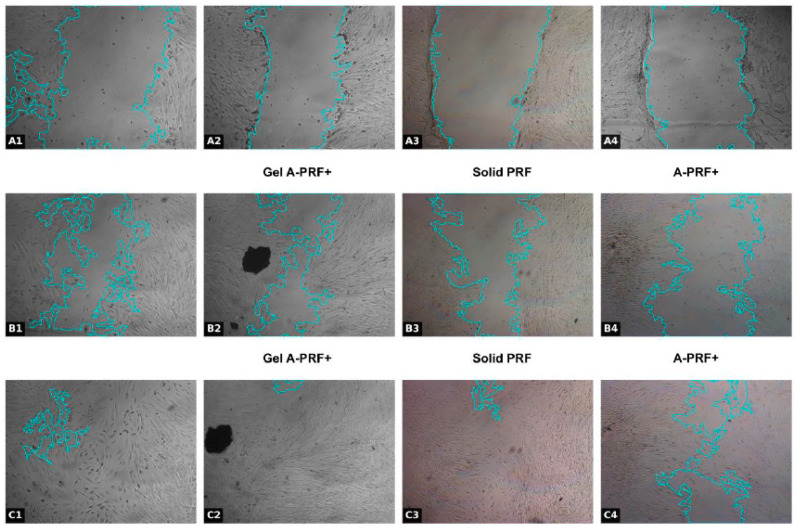
Representative images of wound healing assay at different time points (Day 1: 0 h, 24 h, 48 h) in dental pulp stem cells exposed to various platelet concentrates. Panels: (**A1**–**A4**) Control group at 0 h; (**B1**) Control group, (**B2**) Gel-A-PRF+ group; (**B3**) Solid PRF group; (**B4**) A-PRF+ group at 24 h; (**C1**) Control group; (**C2**) Gel-A-PRF+ group; (**C3**) Solid PRF group; (**C4**) A-PRF+ group at 48 h; Scale bar = 500 µm (applies to all panels).

The results from the first day of the study are presented in Figure 3 and Figure 4 and Table 2.

Image J software (Wound Healing Size plugin for Image J) was used to calculate the wound area in pixels, wound width in pixels, and wound area as a percentage. From these data, we calculated the average values, as shown in Figure 2, which illustrates the dynamics of wound closure in the different PRF groups compared to the control group. When tracking Day 1, it is evident that the quantitative assessment of the images reveals that Gel A-PRF+ yields the most pronounced reduction in wound percentage, area in pixels, and width in pixels, as early as 24 h and especially at 48 h. Solid PRF and A-PRF+, on the other hand, did not reach statistical significance compared to the control group, which highlights the specific effectiveness of Gel A-PRF+.

*p*-values and corrected *p*-values are indicated. At 24 h, a significant difference was found between the Gel A-PRF+ and A-PRF+ groups, and at 48 h, between the Control group and the Gel-A-PRF+ group. This shows that the effect of the studied biomaterials on cell migration varies over time.

According to the results of the present study, we observed a substantial effect size for the tested materials both relative to the control cells and among the materials themselves.

The results obtained are visualized in Figure 4.

### 3.2. Results from the Fourteenth Day of the Study

**Figure 5:**
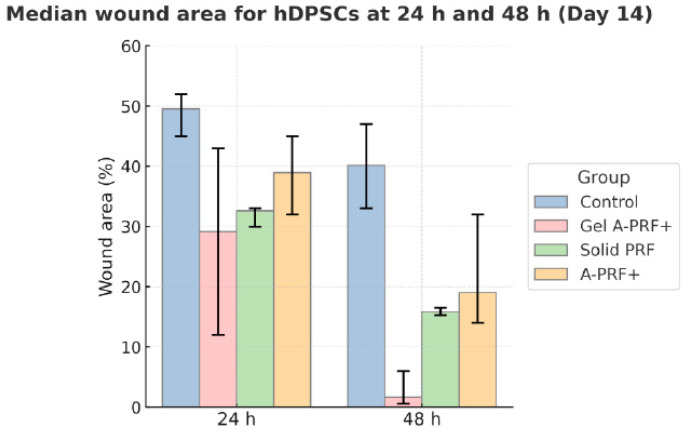
Scratch wound healing assay—Day 14. Median wound area (%, min–max) of hDPSCs cultured with autologous platelet concentrates after 14 days of conditioning. Gel A-PRF+ and Solid PRF induced markedly greater wound closure versus control, confirming the time-dependent enhancement of cell migration under prolonged exposure conditions.

**Figure 6:**
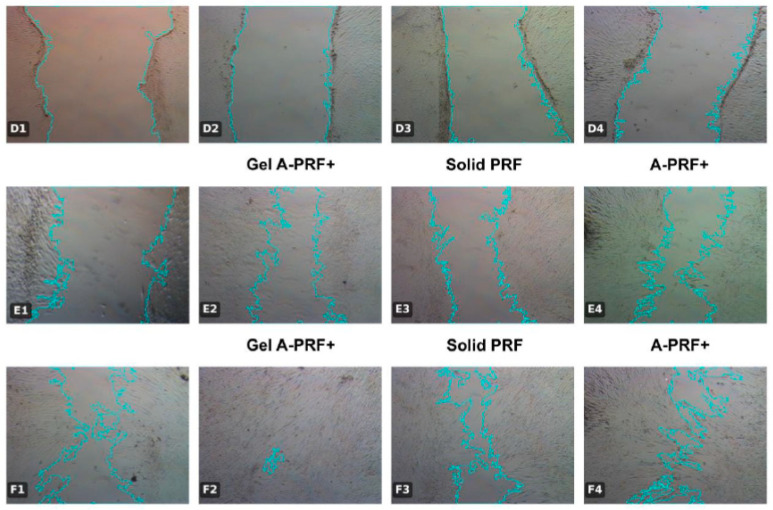
Representative images of wound healing assay at different time points (Day 14: 0 h, 24 h, 48 h) in dental pulp stem cells exposed to various platelet concentrates. Panels: (**D1**–**D4**) Control group at 0 h; (**E1**) Control group, (**E2**) Gel-A-PRF+ group; (**E3**) group; (**E4**) group at 24 h; (**F1**) Control group; (**F2**) Gel-A-PRF+ group; (**F3**) Solid PRF group; (**F4**) A-PRF+ group at 48 h; Scale bar = 500 µm (applies to all panels).

The results from the first day of the study are presented in Figure 5 and Figure 6 and Table 3.

A post hoc Mann–Whitney U test, with the Bonferroni correction for multiple comparisons, revealed statistically significant differences in the percentage of wound area reduction between the control group and the three materials studied. The results are presented in Table 3.

The results are illustrated with photographs of the culture media (Figure 6).

## 4. Discussion

Our results confirm that isolated dental pulp stem cells exhibit characteristic features of mesenchymal human stem cells. The expression of CD44 is consistent with published data, which identify this marker as a reliable indicator of mesenchymal origin [17]. The presence of Nestin and DSPP indicates the potential of hDPSCs for both neurogenic and odontogenic differentiation, which corresponds to their role in regenerative endodontics [18]. CK19 is also expressed, as reported in studies on pulp stem cells [19] and highlighted in a review of oral stem cells [20]. This expression confirms the heterogeneity of the cell population, indicating that these cells have the potential to differentiate into various cell types, contributing to their regenerative capabilities.

Wound healing is a complex physiological process characterized by four main phases: phase (1) hemostasis; phase (2) inflammation; phase (3) proliferation; and phase (4) maturation/remodeling. During hemostasis, activated platelets release growth factors that prepare the environment for the proliferation of mesenchymal stem cells. During the inflammation phase, macrophages release cytokines that direct the migration of mesenchymal stem cells to the damaged site. During the proliferative phase, stem cells migrate to the damaged site, proliferate, and differentiate into various cell types in response to local signals [2].

The scratch wound healing assay is a method for quantitatively assessing cell migration in wound closure in vitro. It is particularly suitable for studying the effect of biological materials such as autologous platelet concentrates, providing a reliable and comprehensive understanding of their impact. It allows tracking the dynamics of wound healing, the time of growth factor release, and their effect on l human dental pulp stem cells (hDPSCs) [21].

Le and Nguyen’s findings, similar to ours, demonstrate the potential of A-PRF+ in accelerating wound healing in the first 1–2 days, albeit with a focus on stem cells from the apical papilla. In contrast, our study, centered on stem cells from human dental pulp, further underscores this potential [22]. Margono et al. also discovered, in line with our results, that A-PRF enhances hDPSC migration in a concentration-dependent manner, with the most significant effect observed at a 10% concentration, a detail that piques our interest due to the rich content of leukocytes and growth factors [14].

This study compares the effects of three autologous blood–derived biomaterials (A-PRF+, Gel A-PRF+, and Solid PRF) on hDPSCs migration in a standardized scratch model. All PRF variants accelerated “wound” closure versus control, with the strongest effect observed for Gel A-PRF+, Solid PRF showing a more sustained effect over time, and A-PRF+ exhibiting a moderate stimulatory response. These findings support the notion that biological conditioning of the milieu—via growth-factor release and cellular cargo (platelets/leukocytes)—is a key mechanism governing early reparative events in the pulp–dentin complex.

The results of our study found that in the early stages—the first day of the study—the overall closure dynamics remained limited, which can be explained by the fact that the release of biomolecules is in its initial stage.

The time dependence (1 day vs. 14 days) reveals a dynamic release/action profile: short conditioning yields a limited effect, whereas prolonged conditioning results in markedly enhanced migration. This aligns with the recognized biphasic kinetics of PRF—an initial burst followed by sustained, lower-intensity release of bioactive mediators—modulated by fibrin network density and cellular load. The stronger early effect of Gel A-PRF+ may reflect higher initial bioavailability of key mediators (e.g., PDGF, TGF-β, VEGF), whereas Solid PRF likely provides a more stable matrix with prolonged release, favoring durable closure. Although we did not perform biochemical/ultrastructural characterization, differences in fibrin architecture and cellular composition among PRF variants plausibly explain the observed effects.

These results can be explained by the observations of Zwittnig et al., who found that PRF matrices release VEGF, PDGF, and TGF-β1 over a period of 7 to 10 days. Solid forms provide a more sustained release, while liquid forms have a faster but shorter-lasting effect. This explains why the most pronounced effect is observed in our hDPSC cultures precisely in continuously conditioned environments (14 days) [23]. Miron et al. confirm that PRF acts as a three-dimensional fibrin matrix, retaining and gradually releasing growth factors over a period of 10 days [15]. This dynamic creates a favorable microenvironment for cell migration and proliferation, which is fully consistent with our results [24]. Jin et al. show that CGF stimulates the proliferation and migration of pulp stem cells at low concentrations, and Chai et al. demonstrate that i-PRF promotes cell migration even in the presence of induced inflammation [7,25].

It is crucial to understand that different PRF forms exhibit varying effectiveness, largely due to their structural features and centrifugation conditions. A-PRF+ is a softer matrix rich in cells, with a rapid release of growth factors, while Solid PRF is a more compact structure that retains larger amounts of biologically active molecules and ensures their slower, yet more prolonged, release. A-PRF+ contains more leukocytes thanks to the low-speed centrifugation concept (Choukroun & Ghanaati, 2018), which contributes to a moderately stable effect [26]. Miron et al. (2020) confirms that different centrifugation protocols lead to significant differences in cell composition and growth factor release dynamics [15]. This underscores the urgency and significance of protocol optimization, as a balance must be found between a rapid initial effect and long-term biological activity [15].

Chen et al. (2020) conclude that AB PRF biomaterials accelerate the healing of difficult wounds [27]. Our study demonstrates the intriguing potential of A-PRF+ to stimulate the migration of pulp stem cells. In the presence of AB biomaterials: A-PRF+, Gel A-PRF+, and Solid PRF, compared to the control group of cells, we observed accelerated healing of experimental wounds, further highlighting the potential of A-PRF+ in regenerative medicine.

Growing literature and clinical interest position PRF not only as a biological adjunct but also as a potential alternative to calcium–silicate materials in vital pulp therapy. Our in vitro data (enhanced hDPSC migration, most pronounced with Gel A-PRF+) are consistent with this trend, but they cannot be interpreted as evidence of clinical equivalence or superiority. Given the distinct mechanisms of action (biological modulation with PRF versus reliable sealing and hard-tissue induction with MTA/Biodentine), prospective randomized designs—testing stand-alone PRF protocols against standard therapy, as well as combined protocols—are required to determine whether PRF can genuinely replace calcium–silicate cements in selected clinical indications [9].

The present study has several limitations that should be acknowledged. First, the experiments were conducted in vitro, which does not fully replicate the complex biological environment of the dental pulp and surrounding tissues. Second, the sample size was limited, and inter-individual variability among donors may have influenced the results. Third, only the migratory behavior of hDPSCs was analyzed, without further assessment of proliferation, differentiation, or the molecular mechanisms involved in tissue regeneration. Finally, no biochemical or ultrastructural characterization of the PRF biomaterials was performed to correlate the content of growth factors or fibrin architecture with the observed biological effects.

Future research should include in vivo models and more detailed molecular investigations to confirm and expand upon the present findings.

## 5. Conclusions

In conclusion, the results of the present study show that the effect of PRF on cell migration is dynamic and dependent on the conditioning time. With short-term conditioning (1 day), the effect is more limited, while prolonged conditioning (14 days) significantly accelerates wound closure. Gel A-PRF+ stimulates rapid, early migration. Solid PRF provides a sustained and long-lasting effect, while A-PRF+ maintains a moderate stimulating effect.

Pulp regeneration is a complex and challenging process. The search for the most promising means of preserving its vitality continues. Based on the present in vitro data, PRF—particularly Gel A-PRF+—may be considered a promising stand-alone candidate for vital pulp therapy. However, available evidence is insufficient to assert a substitutive role; rigorous randomized clinical trials with adequate power and predefined endpoints (e.g., clinical success, reparative dentin formation, retreatment rates) are necessary to validate equivalence or superiority.

## Figures and Tables

**Table 1:** Centrifuge Configuration for PRF: Comparative Summary of Fixed-Angle (Choukroun) and Horizontal (Miron) Systems.

Parameter	Fixed-Angle (“Vertical”, Choukroun)	Horizontal (Swing-Out, Miron)
Rotor orientation	Tubes fixed ~25–45°; separation along an inclined column	Buckets swing to true horizontal; separation across a short vertical column
RCF field	Steeper radial RCF gradient along tube length	More uniform in-tube RCF distribution
Cell distribution	Sharper phase demarcation; higher g/longer spins may drive cells toward RBC layer	Broader interface; often higher platelet/leukocyte recovery at matched RCF/time
Typical PRF protocols *	A-PRF/A-PRF+: lower g, longer time (≈few hundred g for ~8–14 min) → looser fibrin, better cell preservation	Solid/liquid PRF variants: multiple validated protocols across a range of g and durations
Tubes/consumables	Prefer glass/plain, no anticoagulant	Same; tube material/coatings can modulate fibrin polymerization
Temperature/braking	Room temperature; gentle braking; start timing after target speed	Same

* The protocol is different for different centrifuges.

**Table 2:** Post hoc Mann–Whitney U test with Bonferroni correction for multiple comparisons regarding the percentage of wound area reduction on the first day of the study at 24 and 48 h.

Days of Investigation Comparison Between Group	24 h/Day 1			48 h/Day 1		
	*p*-Value Mann–Whitney	p-Bonferroni	Effect Size (r)	*p*-Value Mann–Whitney	p-Bonferroni	Effect Size (r)
Gel-A-PRF+ vs. Control	0.0181	0.1084	0.506 ***	0.0006 *	0.0036 *	0.731 **
Solid-PRF vs. Control	0.067	0.4022	0.394	0.8157	1	0.056
A-PRF+ vs. Control	0.067	0.4022	0.394	0.0001 *	0.0004 *	0.843 ***
Gel-A-PRF+ vs. Solid-PRF	0.1028	0.6168	0.372	0.0252	0.1512	0.507 ***
A-PRF+ vs. Gel-A-PRF+	0.0002 *	0.001 *	0.845 **	0.0002 *	0.0010 *	0.845 **
Solid-PRF vs. A-PRF+	0.011	0.0661	0.575 ***	0.0002 *	0.0010 *	0.845 **

A difference of *p* < 0.05 is considered statistically significant. It is marked with *. Guidelines for effect size: ~0.10 small (*), ~0.30 medium (**), ≥0.50 large effect (***).

**Table 3:** Results of post hoc analysis with Mann–Whitney U test with Bonferroni correction for multiple comparisons regarding the percentage of wound area reduction for the fourteenth day (day 14) of the 24 and 48 h study.

Days of Investigation Comparison Between Group	24 h/Day 14			48 h/Day 14		
	*p*-Value Mann–Whitney	p-Bonferroni	Effect Size (r)	*p*-Value Mann–Whitney	p-Bonferroni	Effect Size (r)
Gel-A-PRF+ vs. Solid-PRF	0.3027	1	0.237	0.0015 *	0.0092 *	0.710 **
Gel-A-PRF+ vs. A-PRF+	0.1028	0.6168	0.372	0.0006 *	0.0033 *	0.778 **
Solid-PRF vs. A-PRF+	0.0042 *	0.0252 *	0.642 *	0.0243	0.1459	0.507 ***
Gel-A-PRF+ vs. Control	0.0006 *	0.0036 *	0.731 **	0.0001 *	0.0004 *	0.843 ***
Solid-PRF vs. Control	0.0001 *	0.0004 *	0.843 ***	0.0001 *	0.0004 *	0.843 ***
A-PRF+ vs. Control	0.0038 *	0.0226 *	0.619 *	0.0038 *	0.0226 *	0.619 *

A difference of *p* < 0.05 is considered statistically significant. It is marked with *. Guidelines for effect size: ~0.10 small (*), ~0.30 medium (**), ≥0.50 large effect (***).

## Data Availability

The data presented in this study are available upon request from the corresponding author.

## Abbreviations

The following abbreviations are used in this manuscript:

AB	Autologous Blood
A-PRF+	Advanced Platelet-Rich Fibrin Plus
CD44	Cluster of Differentiation 44
CGF	Concentrated Growth Factors
CK19	Cytokeratin 19
DAPI	4′,6-diamidino-2-phenylindole
DMEM	Dulbecco’s Modified Eagle Medium
DSPP	Dentin Sialophosphoprotein
EDTA	Ethylenediaminetetraacetic acid
FBS	Fetal Bovine Serum
Gel A-PRF+	Gel form of Advanced Platelet-Rich Fibrin Plus
hDPSCs	Human Dental Pulp Stem Cells
IL-1β	Interleukin-1 beta
IL-4	Interleukin-4
IL-6	Interleukin-6
PBS	Phosphate-Buffered Saline
PDGF	Platelet-Derived Growth Factor
PRF	Platelet-Rich Fibrin
SD	Standard Deviation
Solid PRF	Solid Platelet-Rich Fibrin
TGF-β1	Transforming Growth Factor Beta 1
TNF-α	Tumor Necrosis Factor alpha
VEGF	Vascular Endothelial Growth Factor
